# Hypergraph Contextuality

**DOI:** 10.3390/e21111107

**Published:** 2019-11-12

**Authors:** Mladen Pavičić

**Affiliations:** Center of Excellence for Advanced Materials and Sensors, Research Unit Photonics and Quantum Optics, Institute Ruder Bošković, Zagreb 10000, Croatia; mpavicic@irb.hr

**Keywords:** quantum contextuality, hypergraph contextuality, MMP hypergraphs, operator contextuality, qutrits, Yu-Oh contextuality, Bengtsson-Blanchfield-Cabello contextuality, Xu-Chen-Su contextuality, entropic contextuality

## Abstract

Quantum contextuality is a source of quantum computational power and a theoretical delimiter between classical and quantum structures. It has been substantiated by numerous experiments and prompted generation of state independent contextual sets, that is, sets of quantum observables capable of revealing quantum contextuality for any quantum state of a given dimension. There are two major classes of state-independent contextual sets—the Kochen-Specker ones and the operator-based ones. In this paper, we present a third, hypergraph-based class of contextual sets. Hypergraph inequalities serve as a measure of contextuality. We limit ourselves to qutrits and obtain thousands of 3-dim contextual sets. The simplest of them involves only 5 quantum observables, thus enabling a straightforward implementation. They also enable establishing new entropic contextualities.

## 1. Introduction

Recently, quantum contextuality found applications in quantum communication [1,2], quantum computation [3,4], quantum nonlocality [5] and lattice theory [6,7]. This has prompted experimental implementation with photons [8,9,10,11,12,13,14,15,16,17,18,19], classical light [20,21,22,23], neutrons [24,25,26], trapped ions [27], solid state molecular nuclear spins [28] and superconducting quantum systems [29].

Quantum contextuality, which the aforementioned citations refer to, precludes assignments of predetermined values to dense sets of projection operators, and in our approach we shall keep to this feature of the considered contextual sets. Contextual theoretical models and experimental tests involve additional subtle issues, such as the possibility of classical noncontextual hidden variable models that can reproduce quantum mechanical predictions, up to arbitrary precision [30] or a generalization and redefinition of noncontextuality [31,32]. These elaborations are outside the scope of the present paper, though, since it is primarily focused on contextuality, which finds applications within quantum computation versus noncontextuality, which is inherent in the current classical binary computation. That means that we consider classical models with predetermined binary values, which can be assigned to measurement outcomes of classical observables, which underlie the latter computation, versus quantum models that do not allow for such values and underlie quantum computation. As for the direct relevance of our results to quantum computation, we point out that the hypergraph presented in Figure 2 of Reference [3]—from which the contextual “magic” of quantum computation has been derived—is the kind of hypergraph contextual sets we present in this paper. However, the hypergraph is from a 4-dim Hilbert space, so, we will not elaborate on it in this paper.

We give a pedestrian overview of our approach, methods and results, as well as their background in the last few paragraphs of this introduction, describing the organization of the paper.

A class of state-independent contextual (SIC) [33] sets that have been elaborated on the most in the literature are the Kochen-Specker (KS) sets [34,35,36,37,38,39,40,41,42,43,44,45,46,47,48]. They boil down to a list of *n*-dim vectors and their *n*-tuples of orthogonalities, such that one cannot assign definite binary values to them.

Recently, different SIC sets have been designed and/or considered by Yu and Oh [49], Bengtsson, Blanchfield and Cabello [33], Xu, Chen and Su [50], Ramanathan and Horodecki [51], and Cabello, Kleinmann and Budroni [52]. They all make use of operators defined by vectors that define their sets. You and Oh construct rather involved expression of state/vector defined 3×3 operators that eventually reduces to a multiple of a unit operator while the other authors make use of projectors whose expressions also reduce to a multiple of a unit operator. Therefore, we call their sets the *operator-based contextuality sets* and assume that they form an *operator contextuality class*. All the sets make use of a particular list of 3-dim vectors and their orthogonal doublets and triplets, such that a given expression of definite binary variables has an upper bound which is lower than that of a corresponding quantum expression. The last two References [51,52] also provide us with the necessary and sufficient conditions for an SIC set in any dimension.

The difference between the KS contextuality and the operator contextuality is that KS statistics include measured values of all vectors from each *n*-tuple, while the statistics of measurements are built on values obtained via operators defined by possibly less than *n* vectors from each *n*-tuple.

In this paper, we blend the two aforementioned contextualities so as to arrive at hypergraph one. We consider hypergraphs with 3-dim vectors in which some of those vectors that belong to only one triplet are dropped, as in the observable approach, and generate smaller hypergraphs from them, such that one cannot assign definite binary values to them, as in the KS approach. We call our present approach the McKay-Megill-Pavičić hypergraph (MMPH) approach. MMPH non-binary sets directly provide us with noncontextual inequalities. On the other hand, via our algorithms and programs we obtain thousands of smaller MMPH sets which can serve for various applications as, for example, to generate new entropic tests of contextuality or new operator-based contextual sets.

The smallest MMPH non-binary set we obtain is a pentagon with five vectors (vertices) cyclically connected with 5 pairs of orthogonality (edges). It corresponds to the pentagram from Reference [53], implemented in [15,20,23]. The difference is that the pentagram inequality is state dependent, while the MMPH pentagon inequality is state independent. More specifically, in Reference [53], one obtains a nonclassical inequality by means of the projections of five pentagram vectors at a chosen sixth vector directed along a fivefold symmetry axis of the pentagram. By our method, one gets a nonclassical inequality between the maximum sum of possible assignments of 1, representing classical measurement clicks and the sum of probabilities of obtaining quantum measurement clicks.

Entropic test of contextuality for pentagram/pentagon has been formulated in Reference [54] following Reference [55]. It can be straightforwardly reformulated for the other MMPH non-binary sets we obtained.

The paper is organized as follows.

In Section 2.1 we present the hypergraph formalism and define *n*-dim MMPH set and *n*-dim MMPH binary and non-binary sets as well as *filled* MMPH set. We explain how vertices and edges in an *n*-MMPH set correspond to vectors and their orthogonalities, that is, *m*-tuples (2≤m≤n) of mutually orthogonal vectors, respectively.

In Section 2.2 we give the KS theorem and a definition of a KS set and prove that a KS set is a special non-binary set. In Definition 3 we define a *critical* KS set, that is, the one which would stop being a KS set if we removed any of its edges. Then we introduce known KS sets to compare them with operator defined sets. In particular, we start with Conway-Kochen, Bub, Peres and original Kochen-Specker’s sets. We show that the number of vectors they are characterised with in the original papers and most of the subsequent ones, as well as in books—that is, 31, 33, 33, and 117, respectively—are not critical. That, actually, enables the whole approach presented in this paper. We show that the aforementioned authors dropped the vectors that are contained in only one triplet. If we took all the stripped vectors into account, that is, if we formed filled sets, we would get 51, 49, 57 and 192 vectors, respectively. These sets are critical and the majority of researchers assumed that their stripped versions are critical too and so they did not try to use them as a source of smaller non-classical 3-dim sets.

**Figure 1:**
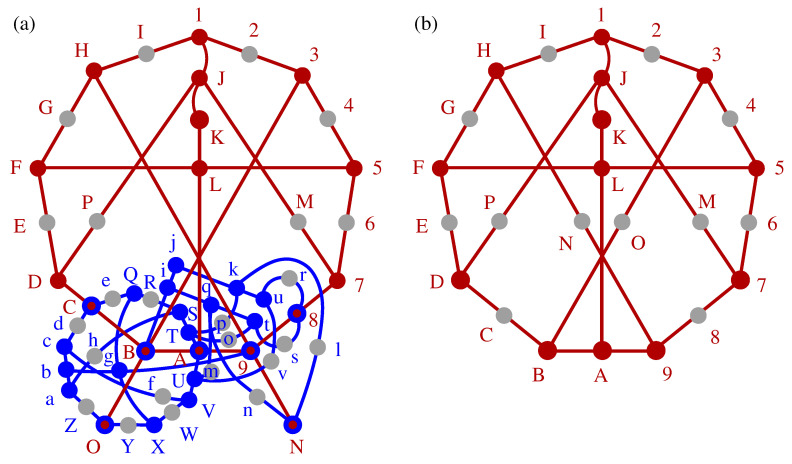
(**a**) Peres’ KS set 57-40 in the MMPH representation and containing the full scale Yu-Oh set (drawn in red); (**b**) The full scale Yu-Oh non-KS set 25–16; Vertices (vectors) that share only one edge (triplet) are given as gray dots. See text.

**Figure 2:**
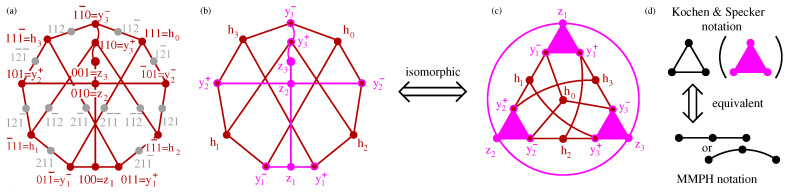
(**a**) An MMPH subgraph of Peres’ KS MMPH; (**b**) Yu-Oh’s reduction of (**a**); (**c**) Yu-Oh’s Figure 2 from [49]; (**d**) Yu and Oh adopted a mixture of Kochen & Specker notation [56]; Cf. (Figure 19 in the [46]), (the triangles in (**c**)) and MMPH notation (the circle in (**c**)).

**Figure 3:**
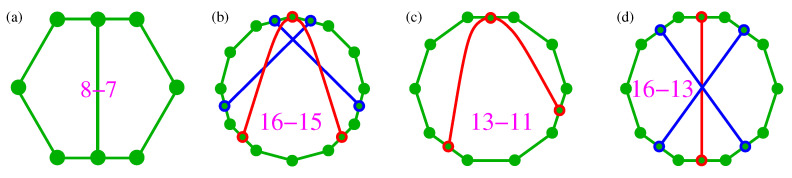
(**a**) Hexagon MMPH from the KS set 192(117)–118 (Figure 6(ii) in the [35]) where it appears in 15 instances; (**b**) a symmetric $\bar{\mathrm{subgraph}}$ of Peres’ MMPH with a non-diagonal $\hat{L}$; (**c**) an asymmetric $\bar{\mathrm{subgraph}}$ of Peres’ MMPH with a diagonal $\hat{L}$ and $\langle \hat{L}\rangle <Max\left[C\right]$; (**d**) a constructed symmetric MMPH with a diagonal $\hat{L}$ and $\langle \hat{L}\rangle <Max\left[C\right]$ but whose full scale version does not have a coordinatization.

Next, we connect and compare KS sets with operator-based sets, in particular YU-Oh’s 13 vector set whose filled version has 25 vectors and 16 triplets—we denote it as 25–16. In Figure 1, we show Yu-Oh’s 25-16 as a subgraph of Peres’ 57–40. In Figure 2, we show how 25–16 can be stripped of vectors contained in only one triplet, so as to arrive at the original Yu-Oh’s 13-16 set. Equations (1)–(6) and their comments explain how Yu and Oh defined their operators with the help of the 13 vectors and how they used them to arrive, via Equation (4), at the inequality defined by Equation (6). We then used the operator expression given by Equation (4) to test 50 sets smaller and bigger than the 13–16 but did not obtain an analogous result. Some of the sets are shown in Figure 3.

In Section 2.3 we give a historical background of stripping the aforementioned vectors that are contained in only one triplet and explain what was behind that “incomplete triplets” issue. Then we give MMPH strings of Conway-Kochen’s 31–37, Bub’s 33–36, Peres’ 33–40 and Kochen-Specker’s 117–118 non-critical but still non-binary non-classical MMPH sets and take them as our master sets from which we generate smaller non-binary critical MMPH sets in the next section. However, we stress that any set we obtain by stripping some other number of vertices contained in only one edge from any one of the original four KS sets can serve us as a master set. We give a Peres’ 40–40 set as an example.

In Section 2.4 we start with a definition of a critical MMPH non-binary set which differs from that of a critical KS set. If we strip more and more edges from a critical KS set we shall never come to a KS set again. This is not so with MMPH non-binary sets. MMPH non-binary critical sets might properly contain smaller MMPH non-binary critical sets whose number of edges is smaller than the original critical set for at least 2 edges.

**Figure 4:**
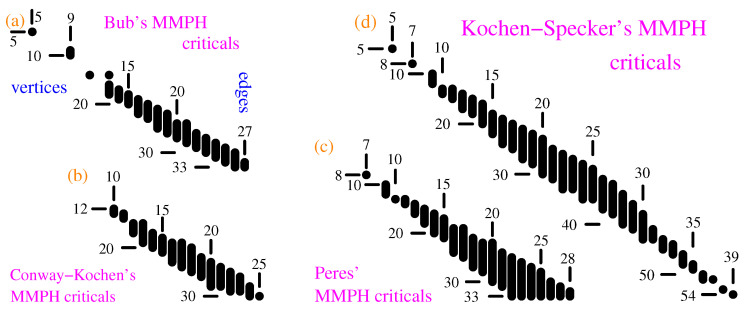
(**a**) Distribution of MMPH non-binary critical sets generated from Bub’s MMPH non-binary master set; (**b**) Conway-Kochen’s criticals; (**c**) Peres’ criticals; (**d**) Kochen-Specker’s criticals.

Via our algorithms and programs, we obtain thousands of critical sets from our master sets, whose distributions are shown in Figure 4. We say that a collection of MMPH non-binary subgraphs of an MMPH master form its class.

Next we define measurements which can distinguish contextual from non-contextual MMPH sets, that is, non-binary from binary ones. Similar to operator-based contextual measurements, dropped vertices are not considered, that is, clicks obtained at their corresponding out-ports are not taken into account when obtaining the statistics of collected data. So, measurements of MMPH non-binary sets are carried out as for KS sets with triplets, that is, with the 1/3 probability of detection at each out-port and via *calibrated* detections of a particle or a photon at out-ports of a gate representing a doublet with the 1/2 probability of getting a click at each of the two considered ports, while ignoring the third one. When a vertex shares a mixture of triplet and doublet edges the probability of detection is 1/p, where 1/3≤p≤1/2. We call detections at all ports notwithstanding whether we include them in our final statistics or not, *uncalibrated* detections—they simply have 1/3 probability of detection at every port.

To obtain contextual distinguishers of an MMPH set we consider the sum of probabilities of getting clicks for all considered vertices and call it a *quantum hypergraph index*. We distinguish a calibrated quantum hypergraph index, which we denote as $HI_{q}$ and an uncalibrated one, which we denote as $HI_{q-unc}$. On the other hand, each MMPH set allows a maximal number of 1s assigned to vertices so as to satisfy the two conditions from Definition 2. We call the number *classical hypergraph index* and denote it as $HI_{c}$. Our *weak* contextual distinguisher is the inequality: $HI_{q}>HI_{c}$ and the *strong* one is the inequality $HI_{q-unc}>HI_{c}$. Yu-Oh, Bub, Conway-Kochen and Peres’ MMPH non-binary sets as well as others given in the section, like, for example, 13–10, satisfy both inequalities.

**Figure 5:**
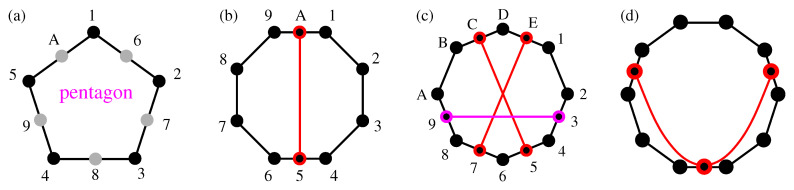
Criticals generated from Bub’s master: (**a**) $\bar{\mathrm{subgraph}}$ pentagon 5–5; (**b**) $\bar{\mathrm{subgraph}}$ 10–9; (**c**) standard subgraph 14–11; Critical generated from Peres’ master: (**d**) 13–11.

**Figure 6:**
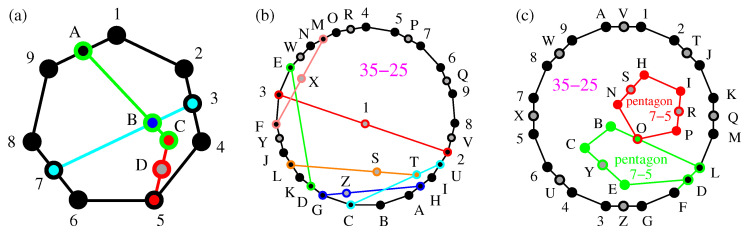
(**a**) Conway-Kochen’s MMPH non-binary critical set 13–10; (**b**) Kochen-Specker’s 35–25a critical with uncalibrated contextuality; the outer loop is a 19–gon; (**c**) Kochen-Specker’s 35–25b critical without uncalibrated contextuality; the outer loop is a 16–gon; See text.

We present several small critical MMPH sets in Figure 5 and Figure 6 and discuss their features. We also calculate Yu-Oh’s inequalities for several sets different from Yu-Oh’s 13–16 set. None of the 50 tested sets satisfy the inequality.

In Section 3 we discuss and reexamine the steps and details of our approach.

## 2. Results

We consider a set of quantum states represented by vectors in a 3-dim Hilbert space $H^{3}$ grouped in triplets of mutually orthogonal vectors. We describe such a set by means of a hypergraph which we call a MMPH. In it, vectors themselves are represented by vertices and mutually orthogonal triplets of them by edges. However, an MMPH itself has a definition which is independent of a possible representation of vertices by means of vectors. For instance, there are MMPHs without a coordinatization, that is, MMPHs for whose vertices vectors one could assign to, do not exist. Also, edges can contain less than 3 vertices, that is, 2 and form doublets. When a coordinatization exist, that does not mean that a doublet belongs to a 2-dim edge but only that we do not take an existing third vertex/vector into account.

### 2.1. Formalism

Let us define the hypergraph formalism.

A hypergraph is a pair v−e where *v* is a set of elements called vertices and *e* is a set of non-empty subsets of *e* called edges. Edge is a set of vertices that are in some sense *related* to each other, in our case *orthogonal* to each other.

The first definition of MMPH was given in Reference [35] where we called them, not hypergraphs, but diagrams. In Reference [46], we gave a definition of an *n*-dim *MMP hypergraph* which required that each edge has at least 3 vertices and that edges that intersect each other in n−2 vertices contain at least *n* vertices. The definition of *MMPH* is slightly different.

**Definition** **1.** *An MMPH is an n-dim hypergraph in which*

*1.*  *Every vertex belongs to at least one edge;*
*2.*  *Every edge contains at least 2 vertices;*
*3.*  *Edges that intersect each other in m—2 vertices contain at least m vertices, where 2≤m≤n.*

Then, in Reference [47] we presented a hypergraph reformulation of the Kochen-Specker theorem [56] from which we derive the following definition of an MMPH non-binary set.

**Definition** **2.** *n-dim MMPH non-binary set, n≥3, is a hypergraph whose each edge contains at least two and at most n vertices to which it is impossible to assign 1s and 0s in such a way that*

*1.*  *No two vertices within any of its edges are both assigned the value 1;*
*2.*  *In any of its edges, not all of the vertices are assigned the value 0.*

*An MMPH set to which it is possible to assign 1s and 0s so as to satisfy the above two conditions we call an MMPH binary set.*

*An MMPH non-binary set with edges of mixed sizes to which vertices are added so as to make all edges of equal size each containing n vertices is called filled MMPH set.*

A coordinatization of an MMPH non-binary set means that the vertices of its *filled* MMPH denote *n*-dim vectors in $H^{n}$, n≥3 and that its edges represent orthogonal *n*-tuples, containing vertices corresponding to those mutually orthogonal vectors. Then the vertices of an MMPH set with edges of mixed sizes inherit its coordinatization from the coordinatization of its filled set. In our present approach a coordinatization is automatically assigned to each hypergraph by the very procedure of its generation from master MMPHs as we shall see below.

In the real 3-dim Hilbert space edges form loops of order five (pentagon) or higher as we proved in Reference [35]. For complex vectors our calculations always confirmed this result but we were unable to find an exact proof. Loops of order two are precluded by Definition 1(3).

MMPH are encoded by means of printable ASCII characters organized in a single string, and within it in edges, which are separated by commas; each string ends with a period. Vertices are denoted by one of the following characters: 1 2 … 9 A B … Z a b … z ! " # $ % & ’ ( ) * - / : ; < = > ? @ [ ∖ ] _ ‘ { | } ~ [35]. When all of them are exhausted one reuses them prefixed by ‘+’, then again by ‘++’ and so forth. An MMPH with *k* vertices and *l* edges we denote as a *k*-*l* set. In its graphical representation, vertices are depicted as dots and edges as straight or curved lines connecting orthogonal vertices. In its ASCII string representation (used for computer processing) each MMPH is encoded in a single line followed by assignments of coordinatization to *k* vertices. We handle MMP hypergraphs by means of algorithms in the programs SHORTD, MMPSTRIP, MMPSUBGRAPH, VECFIND, STATES01, and others [6,35,38,39,57,58].

### 2.2. KS vs. Operator Contextuality

Let us start with the *Kochen-Specker* theorem and KS sets. Then we shall connect them with the vectors and operators of one type of operator-based contextuality introduced by Yu and Oh.

**Theorem** **1.** (Kochen-Specker [56,59,60])**.** *In $H^{n}$, n≥3, there are sets of n-tuples of mutually orthogonal vectors to which it is impossible to assign 1 s and 0 s in such a way that*

*1.*  No two orthogonal vectors are both assigned the value 1;
*2.*  In any group of n mutually orthogonal vectors, not all of the vectors are assigned the value 0.

*The sets of such vectors are called KS sets and the vectors themselves are called KS vectors.*

There is a one-to-one correspondence between KS *n*-tuples of vectors and MMPH edges when they are all of their maximal size, as established in Reference [35,46,47,48] and between KS vectors and MMPH vertices with coordinatization within an MMPH with maximal edges.

**Theorem** **2.** *An n-dim MMPH non-binary set with a coordinatization whose each edge contains n vertices, is a KS set.*

**Proof.**  It follows straightforwardly from the KS theorem, its definition of a KS set and the aforementioned correspondences between its vectors and MMPH vertices. □

In 1988, Asher Peres presented a simple proof of the KS theorem in a 3-dim Hilbert space using real vectors [61]. He implicitly made use of 57 vectors/rays and 40 triplets of mutually orthogonal vectors but seemed to have dropped 24 vectors that appear in only one triplet and called his proof a “33 vector [ray] proof.” However, he admitted the role of the remaining vectors, “It can be shown that if a single ray is deleted from the set of 33, the contradiction disappears. It is so even if the deleted ray is not explicitly listed in table 1.” ([61], L176, bottom paragraph). From Reference ([61], Table 1) we can reconstruct the 33 vectors within their 40 triplets together with the “non-explicit” 24 vectors and represent them in our MMPH notation, obtaining an MMPH non-binary set with 57 vertices (vectors) and 40 edges (triplets), that is, a 57–40 KS set. We did so in two different ways with two resulting (but isomorphic) hypergraphs in Reference ([6], Figure 4) and Reference ([46], Figure 19). Here we give a third MMPH representation (isomorphic to the previous two) which contains the so-called full scale Yu-Oh set 123,345,567,789,9AB,BCD,DEF,FGH,HI1,1JK,KLA,5LF,JPD,JM7,3OB,HN9. we elaborate on below. The representation is carried out via our programs SUBGRAPH and LOOP [47].

Peres’ 57–40 MMPH KS set reads:

123,345,567,789,9AB,BCD,DEF,FGH,HI1,1JK,KLA,JM7,3BO,H9N,JPD,FL5,QRS,STA,AUV,VWX, XYO,OZa,abc,cdC,CeQ,Sha,QgX,Vfc,bg9,qmU,Nnq,Bij,jku,klN,ur8,8st,iqt,Tpk,Tot,uvU.

Its graphical representation is given in Figure 1a.

Notice that gray dots 8,D,N,O in Figure 1b are not gray in Figure 1a and therefore the representation of the original full scale 57–40 Peres KS set (with all gray dots included) by means of the three original Yu-Oh non-KS sets (with gray vertices dropped), as depicted in Figure 1 of [62], apparently does not work. Also, as verified with our program SUBGRAPH, Yu-Oh’s set is not a subgraph of Peres’ 33–40 set (with all gray dots dropped). On the other hand, Yu-Oh’s set cannot be a subgraph of Peres’ 57–40 because it lacks gray dots. The full scale Yu-Oh’s set 25–16 shown Figure 1b is, of course, a subgraph of the full-scale Peres’ 57-40 set as shown in Figure 1a and confirmed by SUBGRAPH.

The arguments that all vertices are indispensable for an experimental implementation of a KS set can be found in Reference ([63], In particular Table on p. 804), Reference ([35], pp. 1583 top, 1588 bottom, and top 1589), and Reference ([64], p. 332, end of the 1st par.). In essence, every *n*-tuple from the KS Theorem 1 should contain no fewer than *n* vectors.

Below, the coordinatization of Peres’ 57–40 set is obtained via VECFIND [47] from the vector components $0,\pm 1,\sqrt{2}$ (the component $-\sqrt{2}$, used by Peres in Reference [61] is not needed):   

1 = {1,$\sqrt{2}$,−1}, 3 = {0,1,$\sqrt{2}$}, 5 = {−1,$\sqrt{2}$,−1}, 7 = {$\sqrt{2}$,1,0}, 8 = {−1,$\sqrt{2}$,0}, 9 = {0,0,1}, A = {0,1,0}, B = {1,0,0}, C = {0,$\sqrt{2}$,1}, D = {0,−1,$\sqrt{2}$}, F = {1,$\sqrt{2}$,1}, H = {$\sqrt{2}$,−1,0}, J = {−1,$\sqrt{2}$,1}, K = {1,0,1}, L = {1,0,−1}, N = {1,$\sqrt{2}$,0}, O = {0,$\sqrt{2}$,−1}, Q = {−1,−1,$\sqrt{2}$}, S = {$\sqrt{2}$,0,1}, T = {−1,0,$\sqrt{2}$}, U = {1,0,$\sqrt{2}$}, V = {$\sqrt{2}$,0,−1}, X = {1,1,$\sqrt{2}$}, a = {−1,1,$\sqrt{2}$}, b = {1,1,0}, c = {1,−1,$\sqrt{2}$}, g = {1,−1,0}, i = {0,1,−1}, j = {0,1,1}, k = {$\sqrt{2}$,−1,1}, q = {$\sqrt{2}$,−1,−1}, t = {$\sqrt{2}$,1,1}, u = {$\sqrt{2}$,1,−1}

The aforementioned Peres’ statement, “if a single ray is deleted from the set of 33, the contradiction disappears” amounts to a coarse definition of a vertex-critical KS set: “A KS [set] is termed critical iff it cannot be made smaller by deleting the [vertices]” [65]. However, in KS sets, there are edges whose removal does not remove any vertex (but nevertheless cause a disappearance of the KS property) and, on the other hand, no vertex can be removed from a KS set without removing at least one edge as well, in the sense that all edges/*n*-tuples should contain *n* mutually orthogonal vertices/vectors.

Therefore, we adopt a definition of an *edge-critical* KS set [6,46,58] (MMPH sets will require a redefinition of critical sets, as we shall see later on):

**Definition** **3.** *KS sets that do not properly contain any KS subset, meaning that if any of its edges were removed, they would stop being KS sets, are called critical KS sets.*

Hence, the set 13,35,57,79,9AB,BD,DF,FH,H1,1JK,KLA,5LF,JD,J7,3B,H9. Yu and Oh obtained in Reference [49] cannot be a KS set since it is a subgraph of a critical KS set (Peres’ set) and therefore cannot provide a proof of the KS theorem contrary to the claim in the title of Reference [49], as we also show in some detail in Reference ([46], Section XII). But, in Reference [49], Yu and Oh do define a new kind of contextuality which we shall analyse and which we summarize as follows.

Consider the MMPH of the Yu-Oh representation of the MMPH Peres’ subgraph, from Figure 1b, shown in Figure 2. They removed all the vertices that share only one edge and which are depicted as gray dots in Figure 2a. Then they define operators by way of the remaining vertices/vectors/rays/states which serve to define filters either for preparation or for detection of arbitrary input or output states, respectively. The procedure goes as follows.

Some of the vectors from Figure 2a are represented as

(1) $$\begin{array}{c} |y_{1}^{-}\rangle =\frac{1}{\sqrt{2}}\left(\begin{array}{c} 0\\ 1\\ -1 \end{array}\right),\;|h_{2}\rangle =\frac{1}{\sqrt{3}}\left(\begin{array}{c} 1\\ -1\\ 1 \end{array}\right),\;|z_{3}\rangle =\left(\begin{array}{c} 0\\ 0\\ 1 \end{array}\right),\;|N\rangle =\frac{1}{\sqrt{6}}\left(\begin{array}{c} 2\\ -1\\ 1 \end{array}\right).\; \end{array}$$

Vectors serve Yu and Oh to define the following operators

(2) $$\begin{array}{c} {\hat{A}}_{i}=I-2|i\rangle \langle i| \end{array}$$

where i=1,…,13 correspond to $y_{1}^{-},y_{2}^{-},\ldots ,z_{3}$ and we add i=14,…,25 corresponding to gray dots in Figure 2a. For instance, for i=1,8,13,20, corresponding to vectors from Equation (1), we have:

(3) $$\begin{array}{c} {\hat{A}}_{1}=\left(\begin{array}{ccc} 1 & 0 & 0\\ 0 & 0 & 1\\ 0 & 1 & 0 \end{array}\right),\;\;{\hat{A}}_{8}=\frac{1}{3}\left(\begin{array}{ccc} 1 & 2 & -2\\ 2 & 1 & 2\\ -2 & 2 & 1 \end{array}\right),\;\;{\hat{A}}_{13}=\left(\begin{array}{ccc} 1 & 0 & 0\\ 0 & 1 & 0\\ 0 & 0 & -1 \end{array}\right),\;\;{\hat{A}}_{20}=\frac{1}{3}\left(\begin{array}{ccc} -1 & 2 & 2\\ 2 & 2 & 1\\ -2 & 1 & 2 \end{array}\right).\; \end{array}$$

The operators can be combined in the following way:

(4) $$\begin{array}{c} {\hat{L}}_{13}=\sum_{i}^{13}{\hat{A}}_{i}-\frac{1}{4}\sum_{i}^{13}\sum_{j}^{13}\Gamma_{ij}{\hat{A}}_{i}{\hat{A}}_{j}=\frac{25}{3}I=8.\dot{3}I, \end{array}$$

where $\Gamma_{ij}=1$ whenever corresponding vectors i,j are orthogonal to each other and $\Gamma_{ij}=0$ when they are not; also $\Gamma_{ii}=0$. The value 25/3 is curious since it is also the sum of probabilities of detecting photons in the full scale setup 25–16 shown in Figure 1b. That may be purely accidental. Also, ${\hat{L}}_{25}$ is not diagonal. Yu and Oh consider neither vectors |i〉 nor operators ${\hat{A}}_{i}$ for i=14,…,25

The fact that each ${\hat{A}}_{i}$ has the spectrum {−1,1,1} prompted Yu-Oh to calculate the upper bound of a corresponding expression for 13 classical variables with predetermined values −1 and 1:

(5) $$\begin{array}{c} C_{13}=\sum_{i}^{13}a_{i}-\frac{1}{4}\sum_{i}^{13}\sum_{j}^{13}\Gamma_{ij}a_{i}a_{j}\leq 8 \end{array}$$

The inequality

(6) $$\begin{array}{c} \langle \hat{L}\rangle >Max\left[C\right] \end{array}$$

has been verified experimentally [16,21] and also improved theoretically by changing the coefficients in Equations (4) and (5) [66,67]. However, no other set, apart from Yu-Oh’s 13–16 itself, with such properties has been found since.

We tested 50 sets and found that $\hat{L}$ of MMPHs without left right symmetry mostly do not have diagonal matrices, although some do, and that $\hat{L}$s of the majority of symmetric MMPHs are also not diagonal; when they are, they are often not multiples of *I*; for the ones whose $\hat{L}$s are multiples of *I* we found that they satisfy either $\langle \hat{L}\rangle <Max\left[C\right]$ or at most $\langle \hat{L}\rangle =Max\left[C\right]$, that is, we have not found instances of Equation (6) being satisfied. We give some examples below.

We should stress here that our definition of a *subgraph* differs from a standard one. The standard definition assumes that a subgraph is a hypergraph contained in a bigger hypergraph as is. In contradistinction, we shall assume that a subgraph might also be a hypergraph obtained from a bigger hypergraph by taking out some edges and connecting the remaining edges together, or simply by taking out some vertices. The latter subgraph we denote as $\bar{\mathrm{subgraph}}$. For instance 123,345,567. is a standard subgraph of 123,345,567,781., while 123,345,561. and 13,345,567,781. are its $\bar{\mathrm{subgraphs}}$. Yu-Oh’s 13–16 set is a $\bar{\mathrm{subgraph}}$ of Peres’ full scale 57–40 set. It is not a subgraph of either Peres’ 57–40 or Peres’ 33–40.

For a symmetric Kochen & Specker’s divided hexagon ([35], Figure 6(ii)) MMPH 8–7, a subgraph of the KS set 117–118 [56], shown in Figure 3a, we obtain $\langle {\hat{L}}_{8}\rangle =Max\left[C_{8}\right]=9/2$. The contextuality of the set has previously been considered in Reference [68].

From Peres’ original KS set, using our programs STATES01, LOOP and VECFIND we can generate arbitrary many subsets. Most of them are asymmetric and their $\hat{L}$s are non-diagonal. Also, many highly symmetric ones, such as, for example, 16–15, shown in Figure 3b with ${\hat{L}}_{16}$ given in Equation (7), are not diagonal.

(7) $$\begin{array}{c} {\hat{L}}_{16}=\frac{1}{6}\left(\begin{array}{ccc} 57 & 4 & 4\\ 4 & 54 & 3\\ 4 & 3 & 60 \end{array}\right) \end{array}$$

An example of a non-symmetric 13–11 with a diagonal $\hat{L}$ is given in Figure 3c. It has $\langle {\hat{L}}_{13}\rangle =7.5$ and $Max[C_{13}]=7.75$, that is, $\langle \hat{L}\rangle <Max\left[C\right]$.

We might try to construct a symmetric MMPH, for example, the 16–13 one given in Figure 3d. For it we obtain $\langle {\hat{L}}_{13}\rangle =9.5$ and $Max[C_{13}]=9.75$, that is, again $\langle \hat{L}\rangle <Max\left[C\right]$. However, the main problem with such constructed MMPHs is that the probability of coming across their filled (full scale) versions with coordinatizations and therefore belonging to the 3-dim Hilbert space is minute, that is, negligible even via automated construction and search on a supercomputer. The full scale version (23–13) of the aforementioned 16–13 apparently does not have a coordinatization either.

We give more examples of $\langle \hat{L}\rangle$ versus Max[C] calculations for other MMPHs in Section 2.4.

### 2.3. MMPH Masters

There are several facts we would like to stress as starting points of our elaboration on the MMPH non-binary sets.

(*i*) Peres wrote, “It can be shown that if a single ray is deleted from the set of 33, the contradiction disappears. It is so even if the deleted ray is not explicitly listed in Table 1.” ([61], L176, bottom paragraph)
Ad (*i*) The first sentence is wrong because MMPH 33–40 set 123,345,47,79,92A,AC,C4,AF,5F,HJ, HL,H7M,NCO,OPQ,QRL,RT,TJ,JPV,VX,XR,Va,La,ce,cT1,cg,FXM,Mhi,ijg,jl,le,ehn,np,pj, nN,gN,t9,tlO,t5,ap1,1MO. is not critical as verified by STATES01. It is also not a KS set but only an MMPH non-binary set. The second sentence is conditionally correct because the full scale MMPH 57–40, 123,345,467,789,92A,ABC,CD4,AEF,5GF,HIJ,HKL,H7M,NCO, OPQ,QRL,RST,TUJ,JPV,VWX,XYR,VZa,Lba,cde,cT1,cfg,FXM,Mhi,ijg,jkl,lme,ehn,nop, pqj,nrN,gsN,tu9,tlO,tv5,ap1,1MO. is a critical KS set but only if assume that with the deleted ray we also delete the edge/triplet it belonged to. (This instance of Peres’ 57–40 KS set is isomorphic to the one given above; the sequence of characters is different due to a reshuffling by automated tools we used to obtain 33–40 as a subgraph of 57–40.
(*ii*) Yu and Oh write, “The KS value assignments to the 13-ray set [13-16] are possible; i.e., no logical contradiction can be extracted by considering conditions 1 and 2 [of Theorem 1].” ([49], p. 3, left column, top)
Ad (*ii*) The claim is provisionally correct, but not because “no logical contradiction can be extracted by considering conditions 1 and 2”—it can be extracted—in 13–16 it is impossible to assign 1s and 0s in such a way that conditions 1 and 2 are satisfied, and not because “value assignments to the 13-ray set are possible”—they are not possible; one cannot assign 1s and 0s to its rays in such a way that conditions 1 and 2 are satisfied—but because the 13–16 set is not a set of triplets and therefore does not satisfy the first part of the KS theorem.

The “incomplete triplets” issue reappears in many papers and books. For instance in Karl Svozil’s book [69] in Section 7.4 there is an excellent symmetric figure of Peres’ 33–40 set [Figure 7.12], we actually made use of to write down MMPH 57–40 set, but we had to add 24 vertices that were not there; 33 vectors and their corresponding logical proposition were explicitly given, but the remaining 24 vectors were not mentioned. In the original Kochen-Specker paper [56] the triplets (edges with 3 vertices) were depicted as triangles and doublets (triplets from which one vertex was dropped) as straight lines—all together 117 vertices of 192 ones contained in 118 triplets. Their triangles are shown in Reference ([35], Figure 6(ii)). The same triangles are used in the Yu-Oh’s set and are shown in Figure 2d. This triangle notation is a source of some confusion in the literature and research, though. For instance, in Reference [52] on p. 4, Figure 1b, where one line from one of the triangles from Yu-Oh’s set is deleted, we read: “(b) $G_{\mathrm{YO}}$ minus one edge.” However, the lines in the triangle are not edges. The whole triangle is an edge (triplet) as shown in Figure 2d. The lines within a triangle are orthogonalities and a removal of one of them means splitting the triplet into two doublets, that is, increasing the number of edges in the set. So, the set in Figure 1a of Reference [52] has 16 edges, while the set in Figure 1b has 17 edges. In any case the set (b) is not a subgraph of (a) nor is (a) a subgraph of (b). Of course, a removal of one of the orthogonalities must also be accompanied by a switch to a new coordinatization of the whole set.

In *The Kochen-Specker Theorem* article in the *Stanford Encyclopedia of Philosophy* only 117 vertices were considered. “[W]hat KS have shown is that a set of 117 yes-no observables cannot consistently be assigned 0-1 values” [70]. Jeffrey Bub writes, “This yields a total of 49 rays and 36 orthogonal triples. Now the only rays that occur in only one orthogonal triple are the 16 rays with a 5 as component. Removing these 16 rays from the 49 rays yields the following set of 33 rays that cannot be colored” [71]. However, 49 rays also cannot be colored and the 49–36 is critical, while 33–36 is not.

These facts offer the following approach, though. The aforementioned conditions 1 and 2 are also contained in the Definition 2 of an MMPH non-binary set and Peres’ 33–40, Yu-Oh’s 13–16, Bub’s 33–36, Conway-Kochen’s 31–37 and Kochen-Specker’s 117–118 sets all violate conditions 1 and 2, thus confirming that these sets are MMPH non-binary sets. Moreover, they actually enable us to get many smaller MMPH non-binary sets from them because none of these sets is critical and they are all equipped with at least the coordinatization they inherit from their full scaled versions 57–40, 25–16, 49–36, 51–37, and 192–118, respectively, but often with even simpler ones.

The MMPH strings of the last three sets are:

Bub’s 33–36 (derived from the full scale 49–36 ([46], Figure 19)): 12,134,156,67,48,9AB,CDE,6B,4E, 2FG,2HI,EG,GB,8I,I7,AJ,AK,C7L,MN9,HON,N3P,PL,MFQ,QL,M5R,RD,DO,STC,JHT,T5U,S3K,SFV,VW, 98W,WU,X9C.

Conway-Kochen’s 31–37 (derived from the full scale 51–37 ([46], Figure 19)): 123,245,26,57,89A,BCD,5D, 3EF,3G,DF,FA,9H,87I,9J,CK,CL,LM,HN,M1N,KO,1OP,Q6R,QGH,BQS, PR,PJ, S4J,SET,NT,TI,RI,UV8, VGK,U6L,4V,UE,18B.

and the Kochen-Specker’s 117–118 (derived from the original full scale 192–118 ([46], Figure 19)): 12,234,45, 56,678,81,9A,ABC,CD,DE,EFG,G9,HI,IJK,KL,LM,MNO,OH,PQ,QRS,ST,TU,UVW,WP,1X, XYZ,Za,ab, bcd,d1,ef,fgh,hi,ij,jkl,le,mn,nop,pq,qr,rst,tm,uv,vwx,xy,yz,z!","u,#$,$%&, &’,’(,()*, *#,e-,-/:,:;,;<,<=>,>e,?@,@[∖,∖],],_‘,‘?,{|,|}~,~+1,+1+2,+2+3+4,+4{, +5+6,+6+7+8, +8+9,+9+A,+A+B+C,+C+5,+D+E,+E+F+G,+G+H,+H+I,+I+J+K,+K+D,?+L,+L+M+N,+N+O, +O+P,+P+Q+R, +R?,37,BF,JN,RV,Yc,gk,os,w!,%),/=,[_,}+3,+7+B,+F+J,+M+Q,95e,HDe,PLe,aTe, mi?,uq?,y’?, ;#?,{]1,+5+11,+D+91,+O+H1,1e?.

All of them have coordinatizations and none of them is critical. They will be our MMPH non-binary *master sets* that we shall get smaller MMPH non-binary critical sets from in Section 2.4. Here, we want to stress that we have chosen the above sets to be our masters for historical reasons. But any set we obtain by stripping the original four KS sets from some other number of vertices being contained in only one edge can serve us as a master set. For instance, by stripping not 24 but 17 such vertices from Peres’ 57–40 KS set, we obtain the following set which we can also use as our master set:

Peres’ 40–40 (derived from the full scale 57–40 ([46], Figure 19)): 123,345,467,78,829,9A,A4,9B,5B,CD,CE, C7F,GAH,HIJ,JKE,KLM,MND,DIO,OPQ,QRK,OST,ET,UVW, UM1,UX,BQF,FYZ,ZaX,ab,bW,WYc,cd,da,cG, XG,e8,ebH,e5,Td1,1FH.

We present two smaller critical MMPH non-binary sets, 35–27 and 38–30, obtained from this 40–40 set, in Appendix A.3 because they are bigger than Peres’ 33–40 and they are critical, while Peres’ 33–40 is not. Also, criticals with 33 or less vertices we obtained from Peres’ 33–40 and from Peres’ 40–40 coincide. The difference is only in criticals with 34 to 38 vertices which we, of course, cannot obtain from Peres’ 33–40 set.

### 2.4. Classes of MMPH Non-Binary Sets, Their Implementation, and Their Inequalities

From the MMPH non-binary master sets given in Section 2.3, we obtain smaller MMPH non-binary critical sets via STATES01. There is a principal difference in the feature of criticality between these sets and the full scale KS sets, though.

If we removed any of the edges of a full scale KS critical set, the remaining set would not be a KS set any more (see Definition 3). If we then continued to strip further edges from the remaining set, we would never arrive at a KS set again. This is not so with an MMPH non-binary critical set. When we remove any of its edges it does stop being an MMPH non-binary set, but if we removed further edges from the obtained set, it would often turn into a smaller MMPH non-binary critical set. Therefore we introduce:

**Definition** **4.** *An MMPH non-binary set is called an MMPH non-binary critical set if a removal of any of its edges would turn the remaining set into an MMPH binary set. MMPH non-binary critical sets might properly contain smaller MMPH non-binary critical sets whose number of edges is smaller than the original critical set for at least 2 edges.*

Bub and Conway-Kochen’s master sets share the coordinatization while Peres and Kochen-Specker’s ones have different ones mutually and with respect to the former two sets. Therefore, also the classes of smaller MMPH non-binary critical sets we obtain from them will be structurally different.

From these master sets we generated classes of smaller MMPH non-binary critical sets by means of our programs [35,47], although the algorithms and programs should be redesigned and rewritten for an automated generation. MMPH sets generated from a master set we call a class of MMPH sets. So, we shall talk about Bub, Conway-Kochen, Peres and Kochen-Specker’s classes. Distributions of their criticals are shown in Figure 4. The criticals are mostly the standard subgraphs of their masters obtained via our automated algorithms and programs, except for a limited number of smaller $\bar{\mathrm{subgraphs}}$ we obtained via new algorithms which are still under development. Most $\bar{\mathrm{subgraphs}}$ have a parity proof unlike most of the standard subgraphs of which only a very few have a parity proof.

Notice that the biggest critical sets in Figure 4a,c have the same number of vertices as their master sets, but 9,12 edges less, respectively.

A possible experimental implementation of MMPH non-binary sets might be made in analogy to the experimental implementation of KS sets carried out in Reference [12]. The difference is that the latter sets contain only triplets, while the former ones contain triplets and doublets, similarly to the Yu-Oh’s 13–16 set, or even only doublets as in the 5–5 set. To carry out measurements on KS sets means that we have to verify that the probability of detecting a particle or a photon at each out-port of a gate representing an edge (triplet) is 1/3. Yu-Oh’s implementation rely on gates defined via Equations (2) and (4) by means of 13 vertices/vectors/rays/states and the gates representing 12 dropped vertices are not considered. Measurements on MMPH non-binary sets might be carried out as for KS sets with triplets (with the 1/3 probability of detection at each out-port) and via calibrated detections of a particle or a photon at out-ports of a gate representing a doublet with the 1/2 probability of detecting a particle at each of the two considered ports. When a vertex share a mixture of triplet and doublet edges the probability of detection is 1/p, where 1/3≤p≤1/2. The data obtained at the out-ports corresponding to the dropped third vertices are discarded or we simply do not measure them at all as in Yu-Oh’s experiments [16,21,66]. To assure an equal distribution of outcomes at each port, the inputs to doublet gates should be scaled up with respect to the full triplet ones by 3/2 and this is why we call them *calibrated*.

The inequalities to be experimentally verified for the MMPH non-binary sets can be defined as for the other two kinds of sets. For instance, for Yu-Oh’s 13–16 set we verify their inequality given by Equation (6): 8.3>8. Let us consider the set as shown in Figure 1b (excluding the gray dots). This set contains 4 triplets and 12 doublets. Vertices A,K,L share only triplets, so the probability of having a click along them is 1/3. Vertices 3,7,D,H share only doublets and the probability of getting clicks along them is 1/2. Vertices 1,5,9,B,F,J share a triplet and two doublets, each, what yields the probability (1/2+1/2+1/3)/3=4/9. Altogether, the probabilities for 13 vertices sum up to 3×1/3+4×1/2+6×4/9=17/3. This sum a quantum hypergraph index of an MMPH set and we denote it as $HI_{q}$. On the other hand, the set 13–16 allows at most four 1s. This is a classical upper bound for getting classical detection clicks, i.e., the maximal number of 1s we can assign to vertices of an MMPH non-binary set so as to satisfy the two conditions from Definition 2, i.e., a classical hypergraph index which we denote as $HI_{c}$. Hence, we obtain the inequality $HI_{q}\left[13-16\right]=17/3=5.\dot{6}>HI_{c}\left[13-16\right]=4$. Notice that even *uncalibrated* probabilities give us $HI_{q-unc}\left[13-16\right]=13/3=4.\dot{3}>HI_{c}\left[13-16\right]=4$. We obtain uncalibrated probabilities by measuring all vertices in all edges in Figure 1b, meaning with gray dots included. With each vertex in every edge we have a probability of getting a click, that is, of assigning 1 to it, being equal to 1/3. If we now selected the 13 red-dot vertices, we would get $13/3=4.\dot{3}$ which is also greater than $HI_{c}\left[13-16\right]=4$. Notice also that the maximal number of 1s we can assign to vertices in the full scale 25–16 set is 11 and that gives us the inequality $HI_{q}\left[25-16\right]=25/3=8.\dot{3}<HI_{c}\left[25-16\right]=11$ which is yet another proof that 25–16 is not a KS set.

It is interesting that three of four considered masters also satisfy the uncalibrated inequality $HI_{q-unc}>HI_{c}$. Bub’s 33–36: $HI_{q-unc}\left[33-36\right]=11>HI_{c}\left[33-36\right]=10$, Conway-Kochen’s 31–37: $HI_{q-unc}\left[31-37\right]=10.\dot{3}>HI_{c}\left[31-37\right]=8$, and Peres’ 33–40 $HI_{q-unc}\left[33-40\right]=11>HI_{c}\left[33-40\right]=6$.

Let us now present several smaller MMPH criticals from each class, consider their properties, and calculate Yu-Oh-like expressions and values for some of them.

The smallest Bub’s critical $\bar{\mathrm{subgraph}}$ with coordinatization we found is the pentagon 5–5 12,23,34,45,51 (with the gray dots excluded) shown in Figure 5a. The full scale hypergraph 10–5 162,273,384,495,5A1 is also shown Figure 5a (with the gray dots included).

As we proved in Reference [35], the smallest loop edges can form in a 3-dim space with vertices endowed with a real coordinatization is a pentagon. We could not find (with Mathematica) a complex coordinatization of any smaller MMPH, either. We conjecture that the filled pentagon MMPH 10–5 is the smallest MMPH with a coordinatization in the 3-dim Hilbert space. Its coordinatization is, for example, 1 = {0,0,1}, 2 = {0,1,0}, 3 = {1,0,1}, 4 = {1,1,−1}, 5 = {1,−1,0}, 6 = {1,0,0}, 7 = {1,0,-1}, 8 = {−1,2,1}, 9 = {1,1,2}, A = {1,1,0}. It, of course, includes the coordinatization of 5–5. As we can easily check, the maximal number of 1s assignable to vertices of 5–5, satisfying the two aforementioned condition, is 2. Thus we have the following contextual inequality $HI_{q}\left[5-5\right]=5\times 1/2=2.5>HI_{c}\left[5-5\right]=2$. Yu-Oh’s approach does not offer us such a contextual distinguisher since for $\hat{L}$ and *C* of Equations (4)–(6) we get ${\hat{L}}_{10}=2.5I$ and $C_{10}\leq 2.5$. Hence, $\langle {\hat{L}}_{10}\rangle =Max\left[C_{10}\right]$. MMPH non-binary $\bar{\mathrm{subgraph}}$ 5–5 can actually be generated in all four MMPH classes, but we have not shown them for Conway-Kochen and Peres’ classes in Figure 4. The pentagon 5–5 has a parity proof.

Subsequent small Bub’s critical $\bar{\mathrm{subgraphs}}$ we obtained, are 9–9 and 10–9. The latter is shown in Figure 5b. Its MMPH string can be easily read from the figure: 12,23,34,456,67,78,89,9A1,A5. Its possible coordinatization is: 1 = {0,0,1}, 2 = {1,1,0}, 3 = {1,−1,1}, 4 = {0,1,1}, 5 = {2,−1,1}, 6 = {1,1,−1}, 7 = {1,0,1}, 8 = {1,2,−1}, 9 = {2,−1,0}, A = {1,2,0}. Vector component ‘2’ is here because the set of 1-A vertex coordinates is a subset of the 1-H set of coordinates of the filled set 17–9. As for the contextuality verification, we have $HI_{q}\left[10-9\right]=6\times \left(1/2+1/3\right)/2+4\times 1/2=9/2=4.5\dot{3}>HI_{c}\left[10-9\right]=4$. On the other hand, we have ${\hat{L}}_{10}=5.5I$ and $C_{10}\leq 5.5$. Hence, $\langle {\hat{L}}_{10}\rangle =Max\left[C_{10}\right]$. The set has a parity proof.

The first standard subgraph in the Bub’s class we found is 14-11 shown in Figure 5c. Its coordinatization is 1 = {2,0,1}, 2 = {−1,−1,2}, 3 = {1,−1,0}, 4 = {1,1,1}, 5 = {2,−1,−1}, 6 = {0,1,−1}, 7 = {2,1,1}, 8 = {−1,1,1}, 9 = {1,1,0}, A = {1,−1,2}, B = {2,0,-1}, C = {1,0,2}, D = {0,1,0}, E = {−1,0,2}. $HI_{q}\left[14-11\right]=4\times 1/3+10\times \left(1/2+1/3\right)/2=11/2=5.5\dot{3}>HI_{c}\left[14-11\right]=5$. The Yu-Oh approach gives: ${\hat{L}}_{10}=8.5I$ and $C_{10}\leq 8.75$. Hence, $\langle {\hat{L}}_{14}\rangle <Max\left[C_{14}\right]$. The set is one of the few standard subgraphs that have a parity proof. The only other Bub’s criticals with a parity proof we found are 14–13, 18–15, 24–19, and 28–23.

Another critical with $\hat{L}=cI$ (*c* is a constant) we found is 14–13: 12,23,34,45,56,67,789, 9A,AB,BC,CD,DE1,E8. $\langle {\hat{L}}_{14}\rangle =7.5<Max\left[C_{14}\right]=7.75$,

Yu-Oh’s 13–16 is from the Peres’ class but the only other critical with $\hat{L}=cI$ we found in Peres’ class is the $\bar{\mathrm{subgraph}}$ 13–11 shown in Figure 4d: 12,234,56,678,89,9A,ABC,CD,D1,35,4B7. $\langle {\hat{L}}_{13}\rangle =7.5<Max\left[C_{13}\right]=7.75$, The coordinatization is 1 = {1,1,$\sqrt{2}$}, 2 = {0,$\sqrt{2}$,−1}, 3 = {0,1,$\sqrt{2}$}, 4 = {1,0,0}, 5 = {1,$\sqrt{2}$,−1}, 6 = {$\sqrt{2}$,−1,0}, 7 = {0,0,1}, 8 = {1,$\sqrt{2}$,0}, 9 = {$\sqrt{2}$,−1,1}, A = {1,0,−$\sqrt{2}$}, B = {0,1,0}, C = {$\sqrt{2}$,0,1}, D = {1,1,−$\sqrt{2}$}. The components $\pm \sqrt{2}$ come from the coordinatization of the filled set 20–11 which requires the components $\pm \sqrt{2},3$, that is, more than Peres’ master set itself. This is because 13–11 is a $\bar{\mathrm{subgraph}}$ and not a standard subgraph of the master set. $HI_{q}\left[13-11\right]=5.5\dot{3}>HI_{c}\left[13-11\right]=5$. The critical 13–11 has a parity proof. We found no standard subgraph of Peres’ master with a parity proof, though.

In Figure 4b, only critical standard subgraphs obtained via automated generation are shown. Hence, they are all subgraphs of Conway-Kochen’s master but we shall explain how one can generate $\bar{\mathrm{subgraphs}}$ from them.

Let us consider Conway-Kochen’s critical 13–10 shown in Figure 6a: 12,234,45,56,678,89,9A1, ABC,3B7,CD5. Its coordinatization is: 1 = {1,1,0}, 2 = {−1,1,1}, 3 = {1,0,1}, 4 = {1,2,−1}, 5 = {0,1,2}, 6 = {1,−2,1}, 7 = {1,0,−1}, 8 = {1,1,1}, 9 = {1,−1,0}, A = {0,0,1}, B = {0,1,0}, C = {1,0,0}, D = {0,2,−1}., after taking into account the filled 17–10 set. Similarly to Yu-Oh’s set, the 13–10 set exhibits both contextual indices: $HI_{q}\left[13-10\right]=4.9\dot{4}>HI_{c}\left[13-10\right]=4$ and $HI_{q-unc}\left[13-10\right]=13/3=4.\dot{3}>HI_{c}\left[13-10\right]=4$. If we take out the vertex D (the gray dot in Figure 6a) the resulting $\bar{\mathrm{subgraph}}$ 12–10 is critical too, which also shows that vertex-criticality is not consistent. Unlike Yu-Oh’s set, neither 13–10 nor 12–10 have $\hat{L}=cI$ satisfied. ${\hat{L}}_{13}$ is not diagonal and ${\hat{L}}_{12}$ is diagonal but it is not a multiple of the unit matrix. The set 12–10 does not exhibit both contextual distinguishers: $HI_{q}\left[12-10\right]=4.75\dot{4}>HI_{c}\left[12-10\right]=4$ but $HI_{q-unc}\left[12-10\right]=12/3=4=HI_{c}\left[12-10\right]=4$. It is, of course, due to the lower number of vertices, since the geometrical structure of the MMPHs, yielding the classical index 4, remains the same.

We find similar features within Kochen-Specker’s MMPH class. Let us take two MMPH criticals from the middle of the distribution shown in Figure 4d. 32–25a: 45,5P7,76,6Q9,98,8V2, 2UI,IHA,AB,BC,CG,GDK,KLJ,JYF,F3,3E,EWN,NMO,OR4,123,DE,STL,UTC,XMF,ZHG. and 35–25b: 12, 2TJ,JK,KQM,ML,LDF,FG,GZ3,34,4U6,65,5X7,78,8W9,9A,AV1,BC,DE,HI,NO,PO,RPI,SNH,YEC,OLB. Their coordinatization is too long to be given here. Neither of them nor any other standard subgraph in the Kochen-Specker’s class we obtained in Figure 4d has a parity proof.

Their different geometrical structure yield different classical hypergraph indices: $HI_{c}\left[35-25a\right]=11$ and $HI_{c}\left[35-25b\right]=12$. However, the number of vertices and therefore the quantum uncalibrated hypergraph indices of both MMPHs are the same: $HI_{q-unc}\left[35-25\right]=35/3=11.\dot{6}$. That means that 35–25a exhibits contextuality even for uncalibrated measurement outcomes, while 35–25a does not. Their calibrated indices are: $HI_{q}\left[35-25a\right]=12.\dot{4}>HI_{c}\left[35-25a\right]=11$ and $HI_{q}\left[35-25b\right]=13.75>HI_{c}\left[35-25b\right]=12$. Pentagons in 35–25b in Figure 6c are subgraphs of Kochen-Specker’s master unlike the pentagon 5–5 (without gray dots) in Figure 5, which is a $\bar{\mathrm{subgraph}}$. If we removed all gray dots, the resulting set 25–25 will not be critical any more, but if we leave S and R in the red pentagon, the resulting 27–25 set will be critical. This cannot be achieved with the green pentagon—leaving Y as the only gray dot in the 26–25 set will not make it critical. $\hat{L}$ of the double pentagon is not diagonal.

In Appendix A we give chosen MMPH non-binary critical sets which are standard subgraphs of the four MMPH master sets.

## 3. Discussion

In the last half a century, a vast number of constructive proofs of quantum contextuality were obtained in even dimensional Hilbert spaces, but only a very few in odd dimensional ones. In particular, in the 3-dim space—Bub, Conway-Kochen, Peres, and Kochen-Specker’s KS sets, Yu-Oh contextual set and Klyachko-Can-Binicioğlu-Shumovsky’s pentagram/pentagon state-dependent set; in total, 6 sets.

In this paper, we present *n*-dim hypergraph contextuality which consists of generating sets which preclude binary assignments of values 0 and 1 to vertices of a hypergraph, such that 1 is assigned to only one of the vertices in each edge of the hypergraph, where an edge can contain less than *n* mutually orthogonal vertices. Such a set which we call an *n*-dim MMPH non-binary set, is defined by Definition 2. We stay with n=3, that is, we deal with qutrits only, although the method can be extrapolated to any dimension. The method serves us to distinguish classical models with predetermined binary values, which can be assigned to measurement outcomes of classical observables underlying classical computation, from quantum models that do not allow for such values and that underlie quantum computation.

Let us make use of a graphical representation of an *n*-dim MMPH to describe the method. Vertices within an MMP hypergraph are drawn as dots and edges containing mutually orthogonal vertices are drawn with the help of straight or curved lines connecting these “orthogonal dots” as shown in Figure 1, Figure 2 and Figure 3, Figure 5 and Figure 6. There can be a different number of vertices/dots in edges. Our program then verifies whether a chosen MMPH *k*-*l* violates or obeys the 0,1 assignment rules from Definition 2. Edges in MMPH *k*-*l* might contain 3 or 2 vertices. We then consider a filled MMPH $k^{\prime }$-*l* in which we add a vertex to each edge which contains only 2 vertices and try to find a coordinatization for it. If successful, we make a one-to-one correspondence between vertices and vectors in the *n*-dim Hilbert space, that is, for the MMPH $k^{\prime }$-*l* set. The MMPH *k*-*l* set inherits the coordinatization from from the MMPH $k^{\prime }$-*l* set. If we implemented the MMPH $k^{\prime }$-*l*, each edge would be a gate with *n* outcomes and the probability of detecting an outcome would be 1/n.

Now, our approach consists of discarding the outcomes corresponding to chosen vertices which share (are contained in) only one edge from chosen edges and considering outcomes only of the remaining vertices. In the 3-dim Hilbert space, that means that some of the edges/gates should be taken as doublets and the others as triplets. Our programs can handle such MMPHs because they are written for edges of mixed sizes. Measurements on MMPH non-binary sets might then be carried for triplets in a standard manner, that is, with the probability of 1/3 of obtaining a click (value 1) at each of the three ports, at a gate corresponding to an edge/triple and via a *calibrated* detection at out-ports of a gate representing a doublet with the probability of 1/2. For vertices that share triplet and doublet edges, the probability would be equal to 1/p, where 1/3≤p≤1/2. Calibration consists of sending three input particles to a doublet gate for each two sent to a triplet gate, that is, the ratio of doublet to triplet inputs should be 3/2.

To obtain a measure of quantum contextuality of an MMPH non-binary set we define hypergraph indices. A classical hypergraph index $HI_{c}$ is the maximal number of 1s we can assign to vertices within edges of an MMPH so as to obey the 0,1 assignment rules from Definition 2. A (calibrated) quantum hypergraph index $HI_{q}$ is the sum of calibrated probabilities for all *k* vertices of the aforementioned *k*-*l* MMPH. An uncalibrated quantum hypergraph index $HI_{q-unc}$ is the sum of 1/3-probabilities for all $k^{\prime }$ vertices of the aforementioned $k^{\prime }$-*l* MMPH. A basic measure of quantum contextuality of an MMPH non-binary set is the inequality $HI_{c}<HI_{q}$. If it were satisfied, the MMPH would be contextual. If not, it would not. A stronger measure of quantum contextuality of an MMPH non-binary set is the inequality $HI_{c}<HI_{q-unc}$. Some of the considered MMPHs do satisfy both inequalities. For instance, Yu-Oh’s set 13–16, MMPH 13–10 shown in Figure 6a, MMPH 35–25a shown in Figure 6b and the MMPH master sets considered in Section 2.4. Other considered critical non-binary MMPHs satisfy only calibrated inequalities but that is sufficient for experimental verification of contextuality and possible applications.

We get thousands of MMPH non-binary sets as follows. For the time being, we start with the previously found KS sets—Bub 49–36, Conway-Kochen 51–37, Peres 57–40, and Kochen-Specker’s 192–118 which are all critical, that is, if we took out any edge from any of them they would stop being KS ([46], Definition 3). However, when we strip all the vertices contained in only one edge we obtain Bub 33–36, Conway-Kochen 32–37, Peres 33–40 and Kochen-Specker’s 117–118 master sets, none of which are critical. This enables us to generate thousands of new smaller MMPH critical sets from them via our programs. Their distributions are shown in Figure 4. Chosen MMPHs critical sets are given in Section 2.4 and Appendix A and shown in Figure 5 and Figure 6. They can be easily implemented, in particular the smaller ones.

The large number of obtained sets can also be used for an automated testing of Yu-Oh’s operators and inequalities along the examples we gave in Section 2.2 and Section 2.4. For that we are developing new algorithms and programs. This is a work in progress.

Next, one can make use of the obtained MMPHs to formulate new entropic tests of contextualities following Kurzyński, Ramanathan and Kaszlikowski [54]. In 2012, they only had one pentagram/pentagon set [53] at their disposal. The pentagon 5–5 set is the simplest MMPH set we obtained (see Figure 4) and many other generated small sets can now serve the purpose.

Also, the methods for evaluating conditions for being a SIC set developed in References [51,52] and the methods of Cabello-Severini-Winter graph-theoretic approach to quantum correlations [72] require samples of hypergraphs and that is what our method offers—a constructive probabilistic generation of arbitrary MMPH sets when coupled with automated vector generation algorithms we developed in Reference [47].

Finally, we stress that the MMPH constructive generation of non-binary quantum sets from operationally chosen vectors out of all possible ones within such sets contribute to our understanding of the physical origin of quantum correlations since they represent a new MMPH *scenario* for getting “quantum correlations from simple assumptions” presented in Reference [73].

## 4. Methods

The methods we use to handle quantum contextual sets rely on algorithms and programs within the MMP language—VECFIND, STATES01, MMPSTRIP, MMPSHUFFLE, SUBGRAPH, LOOP and SHORTD developed in References [6,35,38,39,57,58,74,75]. They are freely available at https://www.irb.hr/users/mpavicic/programs/. MMPHs can be visualized via hypergraph figures consisting of dots and lines and represented as a string of ASCII characters. The latter representation enables the processing of billions of MMPHs simultaneously via supercomputers and clusters. For the latter elaboration, we developed other dynamical programs specifically to handle and parallelize jobs with arbitrary number of MMP hypergraph vertices and edges.

## Abbreviations

The following abbreviations are used in this manuscript:

KS	Kochen-Specker
MMPH	McKay-Megill-Pavičić hypergraph

## Appendix A. ASCII Strings from MMPH Non-Binary Classes

Below we give several chosen standard subgraphs from the four classes of critical MMPH sets shown in Figure 4. The first number in each line is *m* of the biggest *m*-gon loop for the MMPH in the line. The second and third numbers are the numbers of the MMPH vertices and edges, respectively. Three commas “,,,” denote the end of a loop and * behind an ASCII symbol means that the symbol belongs to the loop.

### Appendix A.1. Bub’s Class

10-v18-e13    213,36,6GC,CDB,BH8,89,9I4,45,5EA,A2,,,73*,9*2*,FD*7.

11-v21-e16    213,3A,AHG,GFE,E57,76,6KL,LD8,89,9IC,C2,,,45*,B3*,D*2*,JF*B,H*8*4.

14-v24-e18    12,2L3,34,4KG,GHI,I85,56,6B,BC,CA,A9,9FE,ED,DO1,,,78*,JH*F*,MN7,ND*C*.

13-v27-e20    213,3L4,45,5B,BC,CMN,NOE,E6F,FD9,9A,AJI,IHG,GP2,,,6*7,87,D*3*,KL*H*, QRO*,O*82*,RD*B*.

17-v30-e23    543,3PC,CB,BA,AON,NJ6,67,7KL,L2S,SRI,IH,HTM,MGD,DE,E9,98,8Q5,,,12*3*, FG*,J*5*,P*O*M*,US*Q*,N*F2*.

17-v33-e26    45,5CL,L7E,EF,FG,GBH,HIJ,JN,NRS,SWO,O6P,PMQ,QA2,2V8,89,9TU,U34,,,12*3*, 6*7*,A*B*,C*D,KL*H*,G*D,M*J*,O*H*3*,XU*R*.

### Appendix A.2. Conway-Kochen’s Class

8-v15-e11    12,2E7,78,8D3,34,4C6,65,5F1,,,9AB,B7*6*,A3*1*.

12-v22-e16    67,7GF,FB,B5D,D3,3ME,EC,CK8,89,9HI,I2A,AL6,,,12*3*,45*,A*B*C*,J42*.

14-v26-e19    312,2F,FMN,NL5,596,67,7OJ,JIE,EB,BA,APD,DC,CQH,H3,,,45*3*,89*,G2*, KL*G,I*H*8.

15-v29-e22    12,2RH,HQ3,34,47M,MT9,9A,AJE,ED,DIF,FG,GC,CB,B5N,NS1,,,5*6,7*8,H*I*, KL8,LD*6,OPG*,PN*M*.

17-v30-e24    12,2TD,DH,HRO,O87,76,65,5P4,43,3SJ,JK,K9L,LIM,MQN,NCE,EB,BU1,,,8*9*, AB*,C*D*,FG,I*G,P*GA,Q*J*F.

### Appendix A.3. Peres’ Class

10-v15-e12    12,2A,AC8,87,7D5,56,6B9,94,43,3E1,,,E*C*B*,FE*D*.

14-v19-e16    12,23,34,4E,EGA,A9,9HB,BC,CFD,D8,87,7I6,65,51,,,I*G*F*,JI*H*.

14-v27-e19    12,2QD,DE,E3I,IJK,KM5,56,6L8,87,7PG,GHF,FAB,BC,CR1,,,3*4,9A*,E*C*,NJ*9, OH*4.

20-v35-e27    213,3G,GLM,MNE,EF,FVX,XYU,UP5,5I,IT7,78,89,9S6,6J,JZQ,QHA,AB,BKD,DC, CR2,,,45*6*,H*I*,F*3*,OP*K*,V*Q*L*,T*S*2*,WX*R*.

22-v38-e30    345,5SU,UTH,HI,IR2,2cZ,ZFa,aJW,WVX,XQG,G7,76,6LC,CB,BMD,DE,EYA,A9,98, 8ON,NbK,KP3,,,12*3*,F*G*,J*5*,J*I*,K*E*,P*Q*L*,R*S*M*,a*Y*O*.

### Appendix A.4. Kochen-Specker’ Class

7-v12-e9    12,23,34,456,6A9,987,7C1,,,5*1*,B8*3*.

12-v19-e14    12,2IA,AB,BC8,87,7E5,56,6D4,43,3FG,G9H,HJ1,,,9*A*,H*D*C*.

16-v30-e21    312,2E,EMN,NL8,8RC,CD,D7,76,6GH,HP9,9SA,AB,BQ4,45,5IJ,JO3,,,8*9*3*,F2*, KL*F,TO*B*,UP*D*.

18-v38-e27    34,4VD,DE,ETG,GF,FcO,ON,NHJ,JK,KSC,CB,BZ5,56,6Y9,9A,AX7,78,8W3,,,12, H*I,LM,PQ,RQ,UMI,aR2,bP1,QN*L.

18-v46-e33    56,6a8,87,7e9,9A,AcC,CB,BdD,DE,EbG,GF,FiP,PQ,QYX,XT,THJ,JK,Kh5,,,12, 34,H*I,LM,NO,RS,T*US,VU,WU,ZRO,fI4,gM3,jW2,kV1,T*NL.

12-v54-e39    78,8oV,VW,WgX,XY,YZ,ZfU,UT,TpA,A9,9Ps,sN7,,,12,34,56,BC,DE,FG,HI,JK, LM,N*O,P*Q,RS,ab,cb,dcS,eaR,hK2,iI1,jM6,kOG,lQ5,mC4,nE3,qY*D,rbB,s*LH,s*JF.

## References

[1] Cabello A., D’Ambrosio V., Nagali E., Sciarrino F. (2011). Hybrid Ququart-Encoded Quantum Cryptography Protected by Kochen-Specker Contextuality. Phys. Rev. A.

[2] Nagata K. (2005). Kochen-Specker Theorem as a Precondition for Secure Quantum Key Distribution. Phys. Rev. A.

[3] Howard M., Wallman J., Veitech V., Emerson J. (2014). Contextuality Supplies the ‘Magic’ for Quantum Computation. Nature.

[4] Bartlett S.D. (2014). Powered by Magic. Nature.

[5] Kurzyński P., Cabello A., Kaszlikowski D. (2014). Fundamental Monogamy Relation between Contextuality and Nonlocality. Phys. Rev. Lett..

[6] Pavičić M., McKay B.D., Megill N.D., Fresl K. (2010). Graph Approach to Quantum Systems. J. Math. Phys..

[7] Megill N.D., Pavičić M. (2011). Kochen-Specker Sets and Generalized Orthoarguesian Equations. Ann. Henri Poinc..

[8] Simon C., Żukowski M., Weinfurter H., Zeilinger A. (2000). Feasible Kochen-Specker Experiment with Single Particles. Phys. Rev. Lett..

[9] Michler M., Weinfurter H., Żukowski M. (2000). Experiments towards Falsification of Noncontextual Hidden Variables. Phys. Rev. Lett..

[10] Amselem E., Rådmark M., Bourennane M., Cabello A. (2009). State-Independent Quantum Contextuality with Single Photons. Phys. Rev. Lett..

[11] Liu B.H., Huang Y.F., Gong Y.X., Sun F.W., Zhang Y.S., Li C.F., Guo G.C. (2009). Experimental Demonstration of Quantum Contextuality with Nonentangled Photons. Phys. Rev. A.

[12] D’Ambrosio V., Herbauts I., Amselem E., Nagali E., Bourennane M., Sciarrino F., Cabello A. (2013). Experimental Implementation of a Kochen-Specker Set of Quantum Tests. Phys. Rev. X.

[13] Huang Y.F., Li C.F., Yong-Sheng Zhang J.W.P., Guo G.C. (2002). Realization of All-or-nothing-type Kochen-Specker Experiment with Single Photons. Phys. Rev. Lett..

[14] Huang Y.F., Li C.F., Zhang Y.S., Pan J.W., Guo G.C. (2003). Experimental Test of the Kochen-Specker Theorem with Single Photons. Phys. Rev. Lett..

[15] Lapkiewicz R., Li P., Schaeff C., Langford N.K., Ramelow S., Wieśniak M., Zeilinger A. (2011). Experimental Non-Classicality of an Indivisible Quantum System. Nature.

[16] Zu C., Wang Y.X., Deng D.L., Chang X.Y., Liu K., Hou P.Y., Yang H.X., Duan L.M. (2012). State-Independent Experimental Test of Quantum Contextuality in an Indivisible System. Phys. Rev. Lett..

[17] Cañas G., Etcheverry S., Gómez E.S., Saavedra C., Xavier G.B., Lima G., Cabello A. (2014). Experimental Implementation of an Eight-Dimensional Kochen-Specker Set and Observation of Its Connection with the Greenberger-Horne-Zeilinger Theorem. Phys. Rev. A.

[18] Cañas G., Arias M., Etcheverry S., Gómez E.S., Cabello A., Saavedra C., Xavier G.B., Lima G. (2014). Applying the Simplest Kochen-Specker Set for Quantum Information Processing. Phys. Rev. Lett..

[19] Zhan X., Zhang X., Li J., Zhang Y., Sanders B.C., Xue P. (2016). Realization of the Contextuality-Nonlocality Tradeoff with a Qubit-Qutrit Photon Pair. Phys. Rev. Lett..

[20] Li T., Zeng1 Q., Song X., Zhang X. (2017). Experimental Contextuality in Classical Light. Sci. Rep..

[21] Li T., Zeng Q., Zhang X., Chen T., Zhang X. (2019). State-Independent Contextuality in Classical Light.

[22] Frustaglia D., Baltanás J.P., Velázquez-Ahumada M.C., Fernández-Prieto A., Lujambio A., Losada V., Freire M.J., Cabello A. (2016). Classical Physics and the Bounds of Quantum Correlations. Phys. Rev. Lett..

[23] Zhang A., Xu H., Xie J., Zhang H., Smith B.J., Kim M.S., Zhang L. (2004). Experimental Test of Contextuality in Quantum and Classical Systems. Phys. Rev. Lett..

[24] Hasegawa Y., Loidl R., Badurek G., Baron M., Rauch H. (2006). Quantum Contextuality in a Single-Neutron Optical Experiment. Phys. Rev. Lett..

[25] Cabello A., Filipp S., Rauch H., Hasegawa Y. (2008). Proposed Experiment for Testing Quantum Contextuality with Neutrons. Phys. Rev. Lett..

[26] Bartosik H., Klepp J., Schmitzer C., Sponar S., Cabello A., Rauch H., Hasegawa Y. (2009). Experimental Test of Quantum Contextuality in Neutron Interferometry. Phys. Rev. Lett..

[27] Kirchmair G., Zähringer F., Gerritsma R., Kleinmann M., Gühne O., Cabello A., Blatt R., Roos C.F. (2009). State-Independent Experimental Test of Quantum Contextuality. Nature.

[28] Moussa O., Ryan C.A., Cory D.G., Laflamme R. (2010). Testing Contextuality on Quantum Ensembles with One Clean Qubit. Phys. Rev. Lett..

[29] Jerger M., Reshitnyk Y., Oppliger M., Potočnik A., Mondal M., Wallraff A., Goodenough K., Wehner S., Juliusson K., Langford N.K. (2016). Contextuality without Nonlocality in a Superconducting Quantum System. Nat. Commun..

[30] Barrett J., Kent A. (2004). Noncontextuality, Finite Precision Measurement and the Kochen-Specker. Stud. Hist. Philos. Mod. Phys..

[31] Kunjwal R., Spekkens R.W. (2015). From the Kochen-Specker Theorem to Noncontextuality Inequalities without Assuming Determinism. Phys. Rev. Lett..

[32] Kunjwal R. (2018). Hypergraph Framework for Irreducible Noncontextuality Inequalities from Logical Proofs of the Kochen-Specker Theorem. arXiv.

[33] Bengtsson I., Blanchfield K., Cabello A. (2012). A Kochen–Specker Inequality from a SIC. Phys. Lett. A.

[34] Cabello A., Estebaranz J.M., García-Alcaine G. (1996). Bell-Kochen-Specker Theorem: A Proof with 18 Vectors. Phys. Lett. A.

[35] Pavičić M., Merlet J.P., McKay B.D., Megill N.D. (2005). Kochen-Specker Vectors. J. Phys. A.

[36] Waegell M., Aravind P.K. (2010). Critical Noncolorings of the 600-Cell Proving the Bell-Kochen-Specker Theorem. J. Phys. A.

[37] Waegell M., Aravind P.K. (2011). Parity Proofs of the Kochen-Specker Theorem Based on 60 Complex Rays in Four Dimensions. J. Phys. A.

[38] Megill N.D., Fresl K., Waegell M., Aravind P.K., Pavičić M. (2011). Probabilistic Generation of Quantum Contextual Sets. Phys. Lett. A.

[39] Pavičić M., Megill N.D., Aravind P.K., Waegell M. (2011). New Class of 4-Dim Kochen-Specker Sets. J. Math. Phys..

[40] Waegell M., Aravind P.K., Megill N.D., Pavičić M. (2011). Parity Proofs of the Bell-Kochen-Specker Theorem Based on the 600-cell. Found. Phys..

[41] Waegell M., Aravind P.K. (2012). Proofs of Kochen-Specker Theorem Based on a System of Three Qubits. J. Phys. A.

[42] Waegell M., Aravind P.K. (2013). Proofs of the Kochen-Specker Theorem Based on the N-Qubit Pauli Group. Phys. Rev. A.

[43] Waegell M., Aravind P.K. (2014). Parity Proofs of the Kochen-Specker Theorem Based on 120-Cell. Found. Phys..

[44] Waegell M., Aravind P.K. (2015). Parity Proofs of the Kochen-Specker Theorem Based on the Lie Algebra E8. J. Phys. A.

[45] Waegell M., Aravind P.K. (2017). The Penrose Dodecahedron and the Witting Polytope Are Identical in ${CP}^{3}$. Phys. Lett. A.

[46] Pavičić M. (2017). Arbitrarily Exhaustive Hypergraph Generation of 4-, 6-, 8-, 16-, and 32-Dimensional Quantum Contextual Sets. Phys. Rev. A.

[47] Pavičić M., Megill N.D. (2018). Vector Generation of Quantum Contextual Sets in Even Dimensional Hilbert Spaces. Entropy.

[48] Pavičić M., Waegel M., Megill N.D., Aravind P. (2019). Automated Generation of Kochen-Specker Sets. Sci. Rep..

[49] Yu S., Oh C.H. (2012). State-Independent Proof of Kochen-Specker Theorem with 13 Rays. Phys. Rev. Lett..

[50] Xu Z.P., Chen J.L., Su H.Y. (2015). State-independent contextuality sets for a qutrit. Phys. Lett. A.

[51] Ramanathan R., Horodecki P. (2014). Necessary and Sufficient Condition for State-Independent Contextual Measurement Scenarios. Phys. Rev. Lett..

[52] Cabello A., Kleinmann M., Budroni C. (2014). Necessary and Sufficient Condition for Quantum State-Independent Contextuality. Phys. Rev. Lett..

[53] Klyachko A.A., Can M.A., Binicioğlu S., Shumovsky A.S. (2008). Simple Test for Hidden Variables in Spin-1 Systems. Phys. Rev. A.

[54] Kurzyński P., Ramanathan R., Kaszlikowski D. (2012). Entropic Test of Quantum Contextuality. Phys. Rev. Lett..

[55] Braunstein S.L., Caves C.M. (1988). Information- Theoretic Bell Inequalities. Phys. Rev. Lett..

[56] Kochen S., Specker E.P. (1967). The problem of hidden variables in quantum mechanics. J. Math. Mech..

[57] McKay B.D., Megill N.D., Pavičić M. (2000). Algorithms for Greechie Diagrams. Int. J. Theor. Phys..

[58] Pavičić M., Megill N.D., Merlet J.P. (2010). New Kochen-Specker Sets in Four Dimensions. Phys. Lett. A.

[59] Gleason A.M. (1957). Measures on the closed subspaces of a Hilbert space. J. Math. Mech..

[60] Zimba J., Penrose R. (1993). On Bell Non-Locality without Probabilities: More Curious Geometry. Stud. Hist. Phil. Sci..

[61] Peres A. (1991). Two Simple Proofs of the Bell-Kochen-Specker Theorem. J. Phys. A.

[62] Bengtsson I. Gleason, Kochen-Specker, and a Competition that Never Was. Proceedings of the AIP.

[63] Larsson J.Å. (2002). A Kochen-Specker Inequality. Europhys. Lett..

[64] Held C., Greenberger D., Hentschel K., Weinert F. (2009). Kochen-Specker Theorem. Compendium of Quantum Physics.

[65] Ruuge A.E. (2012). New Examples of Kochen–Specker-Type Configurations on Three Qubits. J. Phys. A.

[66] Cabello A., Amselem E., Blanchfield K., Bourennane M., Bengtsson I. (2012). Proposed Experiments of Qutrit State-Independent Contextuality and Two-Qutrit Contextuality-Based Nonlocality. Phys. Rev. A.

[67] Kleinmann M., Budroni C., Larsson J.Å., Gühne O., Cabello A. (2012). Optimal Inequalities for State-Independent Contextuality. Phys. Rev. Lett..

[68] Clifton R. (1993). Getting Contextual and Nonlocal Elements-of-Reality the Easy Way. Am. J. Phys..

[69] Svozil K. (1998). Quantum Logic.

[70] Held C., Zalta E.N. (2018). The Kochen-Specker Theorem. The Stanford Encyclopedia of Philosophy.

[71] Bub J. (1996). Schütte’s Tautology and the Kochen-Specker Theorem. Found. Phys..

[72] Cabello A., Severini S., Winter A. (2014). Graph-Theoretic Approach to Quantum Correlations. Phys. Rev. Lett..

[73] Cabello A. (2019). Quantum Correlations from Simple Assumptions. Phys. Rev. A.

[74] Pavičić M., Megill N.D., Engesser K., Gabbay D., Lehmann D. (2007). Quantum Logic and Quantum Computation. Handbook of Quantum Logic and Quantum Structures.

[75] Megill N.D., Pavičić M. (2017). New Classes of Kochen-Specker Contextual Sets (Invited Talk). Proceedings of the 40th MIPRO Convention.

